# Plasmonic Effect of Ag/Au Composite Structures on the Material Transition

**DOI:** 10.3390/nano12172927

**Published:** 2022-08-25

**Authors:** Xiaohua Wang, Chengyun Zhang, Xilin Zhou, Zhengkun Fu, Lei Yan, Jinping Li, Zhenglong Zhang, Hairong Zheng

**Affiliations:** 1 School of Physics and Information Technology, Shaanxi Normal University, Xi’an 710119, China; 2 School of Electronic Engineering, Xi’an University of Posts & Telecommunications, Xi’an 710121, China

**Keywords:** Ag/Au composite structures, surface plasmon resonance, photothermal effect, crystal transition

## Abstract

Noble metal nanostructures can produce the surface plasmon resonance under appropriate photoexcitation, which can be used to promote or facilitate chemical reactions, as well as photocatalytic materials, due to their strong plasmon resonance in the visible light region. In the current work, Ag/Au nanoislands (NIs) and Ag NIs/Au film composite systems were designed, and their thermocatalysis performance was investigated using luminescence of Eu^3+^ as a probe. Compared with Ag NIs, the catalytic efficiency and stability of surface plasmons of Ag/Au NIs and Ag NIs/Au film composite systems were greatly improved. It was found that the metal NIs can also generate strong localized heat at low temperature environment, enabling the transition of NaYF_4_:Eu^3+^ to Y_2_O_3_: Eu^3+^, and anti-oxidation was realized by depositing gold on the surface of silver, resulting in the relative stability of the constructed complex.

## 1. Introduction

Plasmon photocatalysis, as a new method to enhance the performance of semiconductor laser catalysis based on localized surface plasmon resonance (LSPR) effect, has attracted great attention in the past decade [1,2,3,4,5]. Compared with conventional thermally driven catalysis, plasmon catalysis can significantly reduce the reaction temperature and achieve the desired catalytic activity in a very short time. More importantly, photocatalysis also shows excellent stability and high selectivity under mild reaction conditions [6]. As a new family of photocatalysts, the catalytic performance of plasmon driven and enhanced photocatalytic and electrocatalytic reactions are highly dependent on the rational design of plasmon nanostructures.

The surface plasmon resonance (SPR) of metal nanostructures can be adjusted by selecting suitable plasmon materials [7,8], particles [9], composites [10], and morphologies [11], et al. As the most common and effective surface plasmon material, Au, Ag and Cu have strong optical adsorption capacity in the visible region [2,6,12]. Au is used for catalysis due to its unique stability and excellent catalytic properties, but it is expensive [13,14]. Ag has advantages with intense electromagnetic field enhancement from a larger extinction cross-section, along with a narrow plasmon linewidth; it is often used as catalyst [15], but the catalytic efficiency of Ag nanostructures gradually weakens with the time stored in air. Thus, the investigation on the plasmon composite structures and their properties that can enhance the advantages and reduce weaknesses of a single element is necessary.

For the study of bimetals, Taerin Chung et al. reported the transfer of metal nanoislands from glass to other different substrates using various dewetting methods, enabling high-throughput and low-cost control and applications of metal nanoislands on different substrates provides direction [16]. Kateryna Loza et al. also used chemical reduction and laser burning methods to obtain the alloy and studied the characterization of the alloy [17]. In these reports, the preparation methods of nanoislands are still relatively complicated, and the influencing factors of plasmonic photothermic effect of nanoislands need further study. Our previous work investigated the photothermal properties of surface plasmon polaritons (SPPs) on metallic NIs by Au [18,19] to induce rapid crystal transitions, which can monitor the local temperature of metal nanoparticles, so here we also use this method to study the plasmonic photothermal effect of bimetallic nanoislands.

In this work, using the method of thermal evaporation, we hope to construct a stable bimetallic nano-island structure, and choose to deposit Au on the Ag surface for the purpose of anti-oxidation. The surface plasmon catalytic effect of Ag NIs and bimetal structures that include the nanoislands formed by Ag/Au (Ag/Au NIs) and Ag NIs covered with Au film (Ag NIs/Au film) were investigated by monitoring the fluorescence of Eu^3+^. It is found that the plasmonic photothermal effect of metal nanoislands can be controlled by the annealing temperature, ambient temperature and the size of the nanoislands. Additionally, compared with Ag NIs, Ag/Au NIs and Ag NIs/Au film present higher catalytic efficiency and better stability, and significant localized heat generated by LSPR of NIs is capable of driving crystal transitions even at low temperature environment. These investigations can provide a better understanding of the surface plasmon catalysis and extend possible applications of metal NIs.

## 2. Materials and Methods

**Figure 1:**
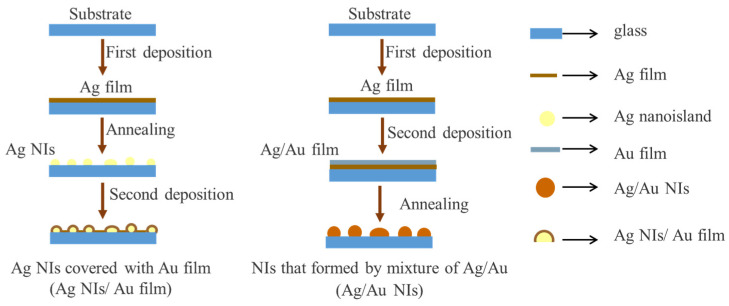
Preparation process of Ag/Au NIs and Ag NIs/Au film.

Ag/Au NIs was prepared on a pre-cleaned glass substrate through high vacuum evaporation. The schematic in Figure 1 shows the preparation procedure of Ag/Au composite NIs structures, in which the annealing was performed in air at RT–400 °C for 30 s. The specific preparation steps were as follows: first, 15 nm Ag film was evaporated on the substrate. Ag NIs/Au film was obtained by annealing the Ag film to obtain Ag NIs, and then a layer of 12 nm Au film was deposited on the surface; then the same annealing treatment was performed. During the evaporation process, the vacuum degree of the vacuum coater was 2.4 × 10^−4^ Pa, and the deposition rate was 0.03 Å/s, and the metal targets were Au wires and Ag nanoparticles with a purity of 99.999%. Polycrystalline NaYF_4_:Eu^3+^ particles were synthesized by wet chemical method. All reagents, including Ln (NO_3_)_3_ (Ln = Y, Eu) (99.9%) and NaF (98%), as well as solvents, were purchased from Sigma-Aldrich Chemicals Co. (Shanghai, China), and used without any further treatment. During the experimental study, the NaYF_4_ particles were evenly spread on the metal film.

## 3. Results and Discussion

**Figure 2:**
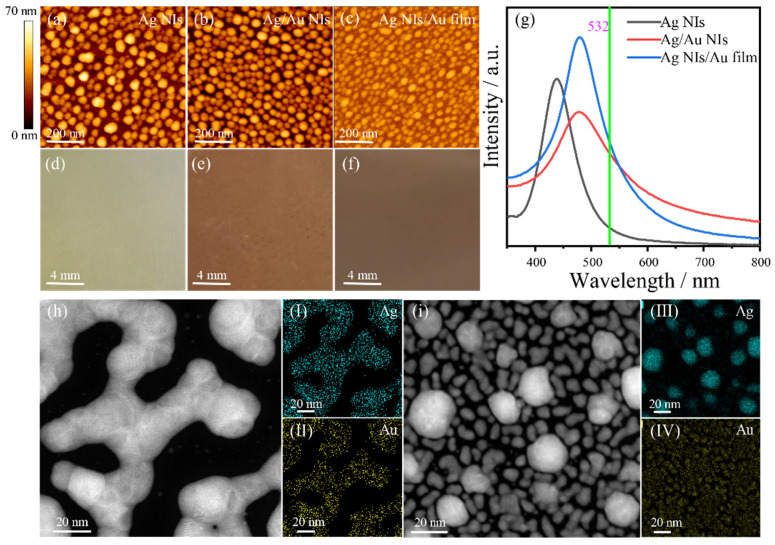
Characterization of metal NIs structures. (**a**–**c**) AFM image, (**d**–**f**) photo and (**g**) absorption spectrum of Ag NIs, Ag/Au NIs and Ag NIs/Au film. (**h**,**i**) HAADF-STEM image, and (**I**–**IV**) EDX elemental mapping of Ag/Au NIs and Ag NIs/Au film.

To investigate the thermocatalytic efficiency of metal NIs film structure, we prepared two types of composite nanosystems, Ag/Au NIs and Ag NIs/Au film, by different deposition and annealing processes. Ag NIs is prepared as the control group that is annealed, at 200 °C, for 30 s. As shown in Figure 2a–f, the AFM characterization results of Ag NIs, Ag/Au NIs and Ag NIs/Au film indicate that the averaged sizes of the island are 25 nm, 20 nm and 30 nm, respectively, and different colors under natural light are presented. In Figure 2g, compared with Ag NIs, the UV–Vis absorption spectra shows that Ag/Au composite nanostructures have a broadened spectral band width and a red-shifted spectral peak position, and the LSPR peak is located at 480 nm, which can better match the irradiation wavelength of 532 nm. The elemental composition of the Ag/Au composite system, Ag/Au NIs and Ag NIs/Au film were determined using a high Angle Ring dark field scanning transmission electron microscope (HAADF-STEM) and the results areas shown in Figure 2h,i. The energy dispersive X-ray (EDX) elemental mapping analysis further demonstrated the microstructure of the composite nanoislands. The characterization results of Figure 2 show that the Ag/Au composite systems have a diverse microstructure compared with Ag NIs, which further leads to its absorption spectrum being adjusted in a wide range.

**Figure 3:**
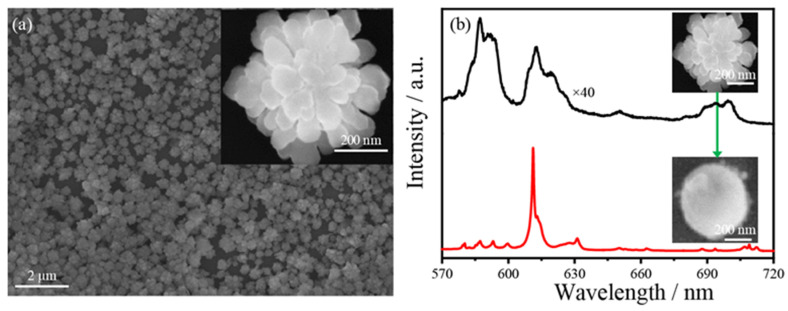
(**a**) SEM images of NaYF_4_:Eu^3+^, the inset is the SEM image after ten times magnification; (**b**) In situ luminescence spectra of Eu^3+^-doped sub-microparticle before and after laser irradiation (23 mW), and inserted SEM images show initial and transformed sub-microparticles, respectively.

Plasmon thermocatalysis of NI films was investigated by observing the transformation of NaYF_4_:Eu^3+^ particles, which was obtained through co-precipitation process. As shown in Figure 3a, the SEM image shows that the product has a flower-like structure, and the overall size is about 500 nm. In the upper right corner is its tenfold magnified SEM image, more detailed sample characterization information is given in Figure S1. The plasmonic photothermal catalysis efficiency of metal NI films were studied by distributing the polycrystalline NaYF_4_ particles uniformly on the NI films and monitoring the spectral changes in the samples under laser irradiation. Figure 3b and Figure S3 show the in situ luminescence spectra of Eu^3+^-doped single sub-microparticle on the metal NI films before and after irradiation with 532 nm wavelength laser. It was found that the luminescence intensity and monochromaticity were greatly improved, and the morphology of the sub-microparticle changed from nanoflower to smoothly spherical particle, of which the image of the sample morphology was located in the upper right corner of Figure 3b. Based on the fluorescence spectra, SEM images, and previous work [20], it is suggested that the Eu^3+^-doped particles after laser irradiation are single crystal spherical Y_2_O_3_:Eu^3+^. To determine the thermal catalysis of metallic NIs, polycrystalline NaYF_4_:Eu^3+^ on a glass sheet were irradiated with laser, and the spectrum did not present any change after 15 min of irradiation with a 23 mW 532 nm laser, as shown in Figure S2. The process by which the transition occurs can be understood as follows: by laser irradiation, the LSPR of NIs is excited and the coherent plasmonic oscillations decay is formed through Landau damping, from which the hot electrons and local heat was generated. Then, these hot electrons can rapidly redistribute energy among low-energy electrons through an electron-electron scattering process. Subsequently, electrons transfer energy to the lattice through electron–phonon coupling, and the equilibrium characterized by high lattice temperature occurs within picoseconds. Due to the high thermal conductivity of NaYF_4_ compared to the surrounding medium (air), the heat generated by the NIs dissipates through the interface with NaYF_4_ nanoflowers through phonon–phonon interactions. Continued thermalization will eventually lead to the temperature equilibrium between NIs and NaYF_4_ within a few nanoseconds. When sufficient heat is delivered to the lattice, NaYF_4_ nanoflowers will begin to transform and finally recrystallize into spherical single-crystallineY_2_O_3_ nanoparticles with minimal specific surface area.

**Figure 4:**
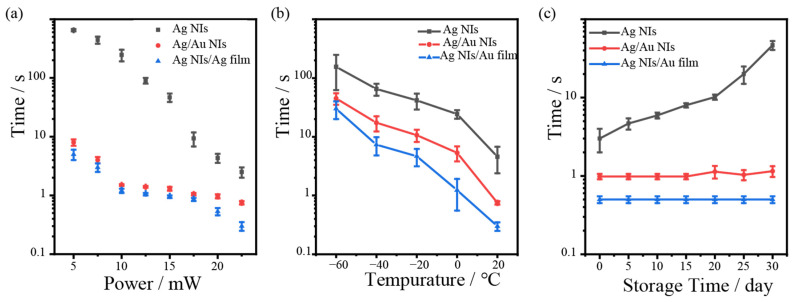
(**a**) Irradiation power dependence on transformation time; (**b**) transformation time at low temperature environment with a laser power of 22.5 mW at 532 nm; (**c**) NI film storage time dependence on the transformation time in air, and the irradiation power is 20 mW laser.

The dynamic process of crystal conversion driven by NIs plasmon can be studied by monitoring the fluorescence emission of Eu^3+^ while controlling the laser irradiation time, and all Nis were obtained by annealing in air, at 200 °C. Firstly, the plasmonic thermocatalytic rate of metal NIs can be regulated by varying the laser radiation power. Figure 4a shows the dependence of irradiation time and power required for the transformation of NaYF_4_ particle into single crystal Y_2_O_3_. As the power increases from 5.0 mW to 22.5 mW, the time required for the crystal transition decreases. For the same irradiation power, Ag NIs, Ag/Au NIs and Ag NIs/Au film catalyze crystal transformation with different rates. Compared with Ag NIs, the crystal transition rate of Ag/Au NIs and Ag NIs/Au film are 4 times and 10 times higher, respectively. At low temperature of 213 K (−60 °C), the metal NI films still present a strong LSP thermal effect, and the generated local heat can also drive the crystal transition. As shown in Figure 4b, when the temperature decreases from 20 °C to −60 °C, although the time required for crystal transformation increases, the transformation can also occur in a short time. In particular, compared with Ag NIs, the stability of the LSPR of the prepared Ag/Au composite NIs system is much better than Ag NIs. As shown in Figure 4c, the transition time also depends on the storage time of the NI films in air. As the storage time increases, the crystal transition time driven by Ag NIs increases. After the one-month storage, the transition time is 17 times that of the original. This is due to the active properties of Ag, which is easily oxidized and reduces the thermal effects of LSPR. However, for Ag/Au NIs and Ag NIs/Au films, the LSPR thermal effect is much more stable even after being stored in the air for one month. It is proved that the Ag/Au composite system can overcome the weakness of the Ag NIs through depositing the Au on the surface of the Ag, which brings the obvious improvement in the catalytic efficiency and stability.

**Figure 5:**
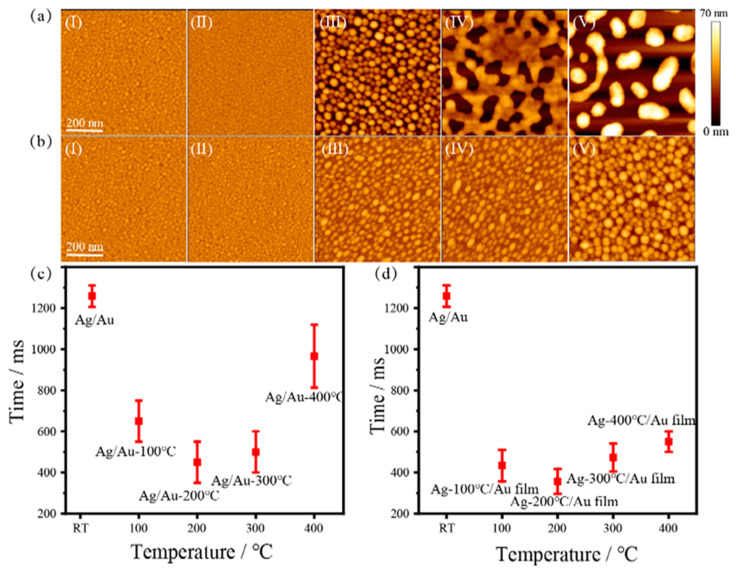
(**a**) Ag/Au NIs and (**b**) Ag NIs/Au films’ AFM images of smooth (**I**) and annealing at 100 °C, 200 °C, 300 °C, 400 °C (**II**–**V**), respectively; (**c**,**d**) are the corresponding nano-island-driven crystal transition times, respectively.

Since the LSPR of metal NIs depends on the geometric properties island size and gap, a series of Ag/Au composite structures were prepared at different annealing temperatures to study the LSPR thermocatalytic efficiency. As shown in Figure 5, the annealing temperature in the range of RT to 400 °C was selected to study the effect of plasmon driven crystal transformation of Ag/Au NIs (a) and Ag NIs/Au films (b). With the increase in temperature, the island particle size and gap increases. Under 532 nm and 22.5 mW laser radiation, the transformation rate of LSPR photothermal drive crystal first increases and then decreases for NIs of I to V in Figure 5, and the fastest transformation was obtained with the NIs III. At the same temperature, the transformation time of Ag NIs/Au film is faster than that of the Ag/Au NIs, as shown in Figure 5c,d. These results suggest that the photothermal catalytic efficiency of LSPR can be controlled by changing the size and gap of plasmonic NIs.

## 4. Conclusions

The plasmonic thermocatalytic effect of metal NIs was investigated by monitoring the transformation rate of the polycrystalline NaYF_4_:Eu^3+^ particle to single crystalline Y_2_O_3_:Eu^3+^ particle. Compared with Ag NIs, Ag/Au NIs and Ag NIs/Au film composite systems present better LSPR stability and thermocatalytic efficiency. It is found that the LSPR thermocatalytic efficiency of metal NIs can be controlled by changing the laser radiation power and morphology of the NIs. Even at a low temperature, NIs can still generate enough amount of heat to drive the crystal transformation. The current study can provide a simple and fast way for the application of Ag plasmon catalysis, which may enable researchers to break the limitation of traditional methods to obtain crystal transition.

## Data Availability

Data are contained within the article or Supplementary Materials.

## Supplementary Materials

The following supporting information can be downloaded at: https://www.mdpi.com/article/10.3390/nano12172927/s1, Figure S1: (a) Transmission electron microscopy (TEM) characterization of NaYF_4_:Eu^3+^ nanoflowers; (b) XRD pattern of NaYF_4_:Eu^3+^ and standard pattern of cubic NaYF_4_. Figure S2: In situ luminescence spectra of NaYF_4_:Eu^3+^ on glass irradiated with 532nm laser irradiation. Figure S3: Evolution of the luminescence spectra for crystal transformation Ag NIs/Au thin film substrate, under 532 nm laser irradiation (22.5 mW at the sample).
