# Supplementary material for: Plasmonic Effect of Ag/Au Composite Structures on the Material Transition
Source: Nanomaterials (Basel). 2022 Aug 25;12(17):2927. doi: 10.3390/nano12172927 (PMC9457859; doi:10.3390/nano12172927)
Supplement: Supplementary file 1 [file nanomaterials-12-02927-s001.zip › nanomaterials-1863191-supplementary.pdf]

nanomaterials

Supplementary Materials

Plasmonic Effect of Ag/Au Composite Structures on the Material Transition

Xiaohua Wang [1], Chengyun Zhang [2], Xilin Zhou [1], Zhengkun Fu [1], Lei Yan [1], Jinping Li [1,*], Zhenglong Zhang [1] and Hairong Zheng [1,*]

[1] School of Physics and Information Technology, Shaanxi Normal University, Xi'an 710119, China
[2] School of Electronic Engineering, Xi'an University of Posts & Telecommunications, Xi'an 710121, China
* Correspondence: ljping@snnu.edu.cn (J.L.); hrzheng@snnu.edu.cn (H.Z.)

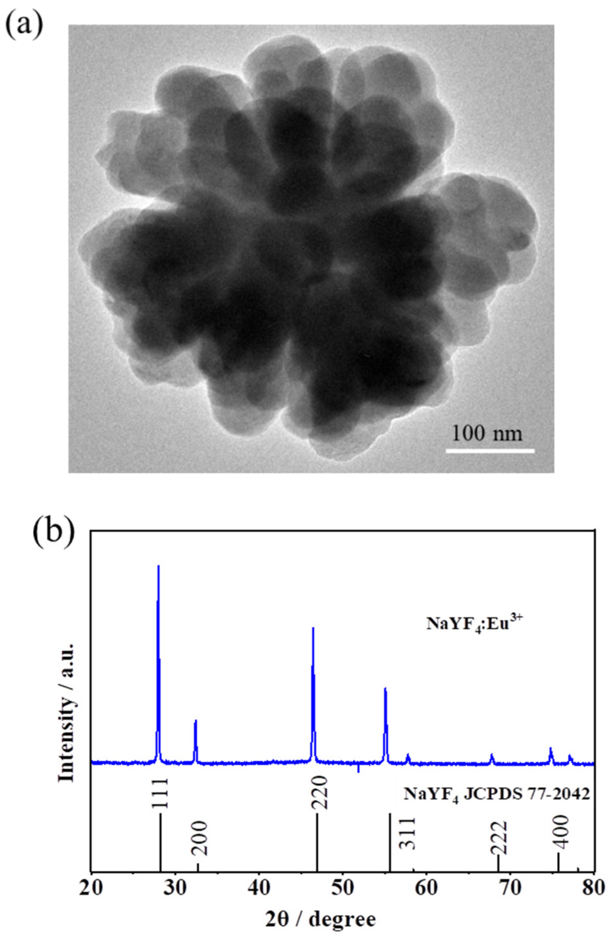

Figure S1. (**a**) Transmission electron microscopy (TEM) characterization of $NaYF_4$:Eu^{3+} nanoflowers; (**b**) XRD pattern of $NaYF_4$:Eu^{3+} and standard pattern of cubic $NaYF_4$.

Citation: Wang, X.; Zhang, C.; Zhou, X.; Fu, Z.; Yan, L.; Li, J.; Zhang, Z.; Zheng, H. Plasmonic Effect of Ag/Au Composite Structures on the Material Transition. *Nanomaterials* **2022**, *12*, 2927. https://doi.org/10.3390/nano12172927

Academic Editor: Vincenzo Vaiano

Received: 28 July 2022
Accepted: 22 August 2022
Published: 25 August 2022

Publisher's Note: MDPI stays neutral with regard to jurisdictional claims in published maps and institutional affiliations.

Copyright: © 2022 by the authors. Submitted for possible open access publication under the terms and conditions of the Creative Commons Attribution (CC BY) license (https://creativecommons.org/licenses/by/4.0/).

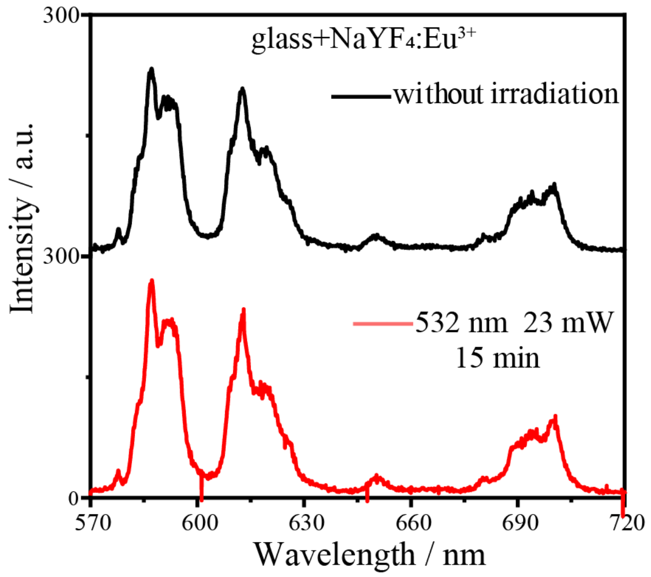

Figure S2. In-situ luminescence spectra of $NaYF_4:Eu^{3+}$ on glass irradiated with 532 nm laser irradiation.

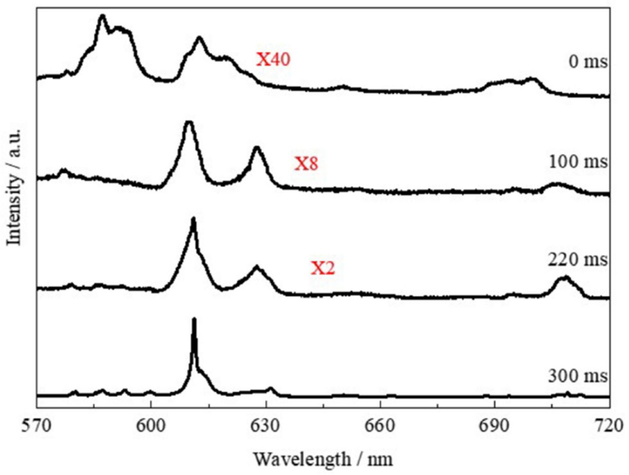

Figure S3. Evolution of the luminescence spectra for crystal transformation Ag NIs/Au thin film substrate, under 532 nm laser irradiation (22.5 mW at the sample).
